# Supplementary material for: Automated estimation of total lung volume using chest radiographs and deep learning
Source: Med Phys. 2022 Apr 18;49(7):4466–77. doi: 10.1002/mp.15655 (PMC9545721; doi:10.1002/mp.15655)
Supplement: Supplementary file 1 — Supporting Information [file MP-49-4466-s001.pdf]

Supplemental Material

Hyperparameter Optimization

A variety of aspects for training a convolutional neural network were considered as hyperparameters; they were learning rate, optimizer, oversampling technique, and data augmentation as seen in **Table S.1.** The random hyperparameter optimization was employed given a predefined range for hyperparameters for each models (frontal CNN, lateral CNN, dual CNN) separately. Augmentation and all the other hyperparameters were also optimized during fine-tuning using the RUMC-real dataset with PFT labels.

Strong augmentation consists of brightness and contrast (factors were chosen uniformly from the range of 0.4 to 1.6) , rotation (degrees range from -15 to 15), translation, and horizontal flip (probability=0.5). Weak augmentation consists of the rotation, horizontal flip, and translation (0.05 for vertical and 0.1 for horizontal maximum absolute fraction), and the degree of augmentations was reduced compared to strong augmentation. Moderate augmentation consists of the same augmentation techniques (and degree ranges) with the weak augmentation, but it additionally includes random resizing. During resizing, the aspect ratio was preserved, and the corresponding TLV reference standard was adjusted accordingly during training. If oversampling hyperparameter was chosen, the chest x-rays with low and high TLV labels, which were underrepresented in the dataset, were oversampled during training. The selected hyperparameters are shown in **Table S.2**

Table S.1: The ranges of the hyperparameters that were defined for the optimization procedure.

Learning rate	$[10^{-5}, 0.001]$
Optimizer	[Adam, SGD]
Data augmentation	[strong, moderate, weak]
Oversampling	[Yes, No]

Table S.2: The final hyperparameters that were selected for the best models on each dataset.

Dataset	Optimizer	Data augmentation	Oversampling	Learning rate
COPDGene-sim	Adam	weak	no	0.00018
RUMC-sim	Adam	weak	yes	2.78x10^{-5}
RUMC-real (CT labels)	Adam & Adam	weak & strong	no & no	0.0001&0.0001
RUMC-real (PFT labels)	Adam	strong	yes	2.6x10^{-5}

The selected hyperparameters of the best models in each experiment. The best model for the RUMC-real (CT labels) is an ensemble model, therefore frontal & lateral model hyperparameters are shown, respectively.

Evaluation Metrics

Mean absolute error (MAE) and mean absolute percentage error (MAPE) were used to evaluate the accuracy of the total lung volume prediction with respect to the reference standard. MAE is formulated as follows:

$$\mathrm{MAE} = \frac{1}{N}\sum_{t=1}^{N} |\boldsymbol{y}_t - \widehat{\mathbf{y}}_{\mathbf{t}}|$$

where $\boldsymbol{N}$ denotes the number of images, $\boldsymbol{y}_t$ represents the reference standard TLV, and $\widehat{\mathbf{y}}_{\mathbf{t}}$ represents the predicted TLV.

MAPE is formulated as follows:

$$\mathrm{MAPE} = \frac{\mathbf{1}}{\boldsymbol{N}}\sum_{\boldsymbol{t}=\mathbf{1}}^{\boldsymbol{N}} \left| \frac{\boldsymbol{y}_t - \widehat{\mathbf{y}}_{\mathbf{t}}}{\boldsymbol{y}_t} \right|$$
